# Supplementary material for: Are carbon emissions trading and green financial instruments synergistic? -Comprehensive quantitative research based on content analysis
Source: PLoS One. 2024 Mar 7;19(3):e0298601. doi: 10.1371/journal.pone.0298601 (PMC10919601; doi:10.1371/journal.pone.0298601)
Supplement: S1 Data — (ZIP) [file pone.0298601.s001.zip › Data/地级市Z_F工作报告.zip!/║■▒▒/╛ú╓▌╩╨/└┤╫╘╣1⁄2╓┌║┼í╛┬φ┐╦╩2╛▌═°í┐_2008.docx]

各位代表：

现在，我代表市人民政府向大会作工作报告，请予审议，并请市政协各位委员和列席人员提出意见。

一、2007年工作回顾

刚刚过去的一年，在省委、省政府和市委的领导下，在市人大、市政协的监督支持下，市政府认真学习贯彻党的十七大精神，以科学发展观统领全局，紧紧依靠广大干部群众，扎实工作，锐意进取，全面超额完成市三届人大三次会议确定的目标任务，经济社会实现又好又快发展。

经济发展态势良好。预计全市生产总值500亿元（当年价），按可比价格增长10.5%以上，人均生产总值突破1000美元。工业经济提速增效。预计全市规模以上工业完成增加值120亿元，增长20%以上，为建市以来最好水平，顺利实现工业兴市五年翻番目标。规模以上企业新增137家，达到766家；工业完成投资90亿元、增长59%，入库税金16亿元、增长32%。满园工程全面推进。全市15个工业园区新增基础设施投资7.4亿元、入园项目245个，其中荆州开发区引进千万元以上项目80个、建成投产65个。产业素质逐步提高。全市产销过10亿元的产业集群新增3个、达到8个。省级以上高新技术企业新增11家、达到67家，新增中国名牌4个、湖北名牌4个，分别达到6个和56个。农村经济持续健康发展。预计粮、棉、油产量分别达到355.8万吨、15.4万吨和48.7万吨，生猪出栏343.3万头，水产品产量75万吨。农业专业化、规模化发展加快。新增畜禽养殖小区35个，建成市级以上水产板块10个；新增市级以上龙头企业38家、农民专业合作社48家、标志性农产品125个。新农村建设稳步推进。发放补贴资金3.67亿元、增1.24亿元，农民负担与上年基本持平；开展新农村建设试点，完成血防整县推进和四湖流域综合治理工程投资12亿元，通村公路、“一建三改”、安全饮水和土地整理等项目建设步伐加快，农村生产生活条件进一步改善。文化旅游资源开发力度加大。熊家冢楚墓、谢家桥1号墓考古发掘与保护取得阶段性成果，启动关公祠、关庙等景点开发工程，实施城区部分景点一票制，全年接待游客511万人(次)，增长18.6%。消费市场更加活跃。制定中心城区商业网点发展规划，推进农村“万村千乡”市场工程，预计社会消费品零售总额290亿元以上，增长17%。金融服务成效显著。金融机构开展满园工程和县市行服务活动，贷款投放大幅增长，预计比年初增40多亿元，全系统实现盈利。财政收入较快增长。实现财政收入34.1亿元、增长17.3%，按可比口径增长24.6%；地方一般预算收入15.2亿元、增长18.5%。国税、地税分别完成收入17.7亿元和10.8亿元，增长30%和21.7%。

改革开放力度加大。加强国有资产监督管理，大力推进国有存续企业资产处置和改制煞尾工作，妥善安置职工，天发集团风险处置取得重大突破，天发石油、天颐科技股权重组顺利完成，国有“非工”企业改革加快推进。农村综合配套改革继续深化，“以钱养事”机制进一步完善，集体林权制度改革稳步实施。调整中心城区财政管理体制，完善土地收购储备制度，明确沙市区与荆州开发区管理范围，城区呈现竞相发展势头。部门预算稳步推进，收支两条线管理得到加强。公务员登记和工资套改基本完成，事业单位改革力度加大，市直减少事业编制538名。深化行政审批制度改革，发展环境进一步优化。招商引资成效明显，全年到位资金100亿元、增长33.3%；利用外资1亿美元、增长15%，新批外资企业25家。海关、出入境检验检疫、口岸等大通关服务效率提高，预计出口4.7亿美元、增长53.6%；楚源、沙隆达等12家企业出口过千万美元、新增6家；盐卡港集装箱吞吐量达到5.2 万标箱，增长30%。

固定资产投资再创新高。全社会固定资产投资突破200亿元，增长33%以上。水利完成投资6.6亿元，四湖流域综合治理力度加大，洪湖围堤一期工程和西干渠疏挖土方任务基本完成，荆南四河和洈水水库加固等重点工程完成年度投资任务。交通完成投资22亿元，东荆河大桥实现主体合龙，随岳高速荆州段、荆岳长江公路大桥、荆监一级公路、盐卡港二期扩建工程建设进展顺利。电力、电信分别完成投资6.6亿元和1.3亿元。中心城区城市建设完成投资3.2亿元，黑化升级荆中路、荆南路、屈原路、迎宾路、公园路和江津路东段，维修改造长港路、航空路，实施荆沙大道续建工程，加强污水管网和绿化等配套设施建设，整治违章建筑和占道经营，城市面貌逐步改善。引导房地产业健康发展，推进危旧房改造，预计完成投资19亿元。加快县市城区和中心镇建设，洪湖瞿家湾镇和监利程集镇被列入全国第三批历史文化名镇，全市城镇化率达到35.1%。

和谐荆州建设扎实推进。加大科技创新力度，争取国家、省级科技投入2108万元，获国家科技进步二等奖1项、省科技进步奖17项。认真落实农村义务教育经费保障机制，投入“两免一补”、中小学危房改造等资金3.1亿元，资助贫困生18万人。推进市直3所中专、5所技校合并重组，职业教育和高等教育办学规模扩大。积极发展文化、广电、体育事业，荆州花鼓戏《渔妈莲妹红军哥》获八艺节创作奖，有线电视通村率达到80%，荆州成功入选奥运火炬传递城市。社会科学、文学艺术、新闻出版等取得新成绩。扩大新型农村合作医疗覆盖面，农民参合率达到78%。加强乡村卫生服务一体化管理和社区卫生服务体系建设，公共卫生服务水平不断提高。认真落实计生政策，符合政策生育率为96.8%，出生人口性别比下降2个单位值。加强基本农田保护与建设，土地整理新增耕地1.5万亩。积极开展节能降耗和环境保护工作，实施20个节能改造项目，采取果断措施，依法关闭宏云、宽宏等23家小造纸企业和3家小水泥厂，治理庙湖污染，启动护城河生态补水工程，红光污水处理厂开始正常运行，洪湖拆围和渔民安置任务顺利完成，万元生产总值综合能耗下降4.6%，化学需氧量排放下降5%以上，二氧化硫减排完成年度任务。深入开展群众性精神文明创建活动，推进公民思想道德建设，涌现了全国道德模范赵传宇、全国十大杰出检察官马俊镠、见义勇为模范家庭杨振环等一批先进人物。加强社会治安综合治理，扎实推进“平安荆州”建设，社会秩序基本稳定。全省社会治安综合治理、关停小造纸和文明新村创建工作现场会在我市召开。健全领导包案和部门联动接访机制，妥善解决信访突出问题。抓好重点领域安全隐患排查整改，强化食品、药品市场监管，重特大安全事故得到遏制。加强国防教育、国防动员、人民防空、预备役部队和民兵建设，认真做好转业退伍军人安置和拥军优属工作，军政军民关系进一步密切。妇女儿童和未成年人、老年人、残疾人的合法权益得到保护。编制、统计、外事侨务、台湾事务、民族宗教、气象、地震、档案、地方志和无线电管理等工作都有新进步。

认真执行人大及其常委会的决议，主动接受法律监督和工作监督，接受人民政协和各民主党派、工商联、人民团体、无党派人士的民主监督，接受新闻舆论和社会公众的监督，认真办理人大代表、政协委员的建议、提案、批评和意见。认真落实源头治腐“四项制度”，扎实开展行风评议和绩效考核，强化行政监察、审计监督和政务督查，坚决纠正损害群众利益的行为，清退教育违规收费500多万元，兑现农民工拖欠工资1030万元。积极推进政务公开，加强决策咨询、电子政务、新闻发言人制度和应急机制建设，政府工作透明度和公共服务能力得到增强。

人民生活水平明显提高。预计城乡居民收入分别突破1万元和4100元，增加2000多元和600多元。开展统筹城乡就业试点，城镇登记失业率控制在4.4%以内。社保扩面新增15万人次，实现收入16亿元。实施农村低保制度，城乡享受低保人数分别达到14万人和12万人，发放低保资金1.25亿元。完善大病医疗和困难救助等制度，发放救助和救灾资金2715万元、水库移民直补资金3015万元。投入扶贫开发资金6600万元，2.8万人实现脱贫。

年初确定的十件实事顺利完成。城镇就业人员新增8.5万人，培训农村劳动力4.6万人、转移14万人；市、县组建惠民医院3所、惠民窗口4个，新增参合农民110万人，筹集大病医疗救助基金1491万元，救助12.5万人次；新建通村公路1700公里，新增通油路、水泥路行政村750个，建成农村公路候车亭200个；改造和新建标准化农家店1048家，新增农村有线电视用户11.6万户，援建村级图书室200个；新增安全饮水受益人口50.5万人，新建沼气池10.8万口，改厕11.2万座，免费救治晚血病人2936人；健全法律援助中心9个，改造乡镇（街道）法律援助办公场所126处；减免中心城区低保家庭学生学杂费、书本费67万元；城区200套廉租房主体工程完工，减免2127户低收入家庭租住国有公房租金49.9万元；完成荆中路等升级改造和南环路路灯建设；免费为250名特困残疾人装配假肢和提供轮椅，为321名特困白内障患者实施复明手术。

各位代表！过去一年的成绩来之不易，是省委、省政府和市委正确领导，全市人民团结拼搏、共同努力的结果。在此，我代表市人民政府，向为荆州改革发展稳定做出积极贡献的广大干部群众，向全市人大代表、政协委员、各民主党派、工商联、人民团体和无党派人士，向人民解放军、武警官兵和政法干警，向所有关心、支持荆州建设与发展的海内外朋友，致以衷心的感谢和崇高的敬意!

在看到成绩的同时，我们也清醒地认识到，发展不快、发展不够仍然是荆州面临的主要问题，政府工作还有不少薄弱环节，经济社会发展中还存在不少困难和问题。具体表现在：工业发展总量不大、结构不优，县域经济、民营经济实力不强，承接产业转移力度不够；资金、土地、环境制约更加明显，体制创新、结构调整、转变经济发展方式等任务艰巨；农业基础脆弱，农村基础设施建设和公共事业发展滞后，农民增收的长效机制尚不健全；就业再就业压力较大，社会保障体系不够完善，食品等价格上涨较快，部分低收入群体的生活还比较困难；社会治安、城市管理、投资环境以及政府自身建设有待进一步改善。对此，我们将高度重视，采取切实有效措施认真加以解决。

二、2008年工作建议

2008年是贯彻落实党的十七大精神的第一年，是实施“十一五”规划承上启下的关键一年，同时，举世瞩目的奥运圣火将在荆州隆重传递，认真做好政府各项工作，意义十分重大。

2008年政府工作的指导思想是：认真贯彻落实党的十七大精神，牢固树立科学发展观，继续解放思想，以大开放为先导，着力加快发展步伐和转变发展方式，着力推进各项改革和创新体制机制，着力改善民生和促进社会事业协调发展，着力加强资源节约和保护生态环境，加快新型工业化、新农村建设和城镇化进程，努力实现经济社会又好又快发展。主要预期目标是：地区生产总值增长11%，万元生产总值综合能耗下降4.6%，化学需氧量排放下降2.5%；固定资产投资增长20%，社会消费品零售总额、外贸出口和实际利用外资分别增长15%，财政收入增长16%；城镇居民人均可支配收入增长10%，农民人均纯收入增长8%，城镇登记失业率控制在4.4%以内，居民消费价格涨幅控制在4.8%以内，人口自然增长率控制在5.5‰以内。为实现上述目标，重点做好以下八个方面的工作。

（一）深入推进工业兴市战略。坚持工业兴市主战略不动摇，狠抓招商引资和项目建设，继续加大投资强度，加快工业园区化、集群化步伐，壮大支柱产业和骨干企业，提升工业总量和产业层次，确保全市规模以上工业增加值增长18%以上。

着力实施满园工程。进一步完善园区规划，科学确定产业发展方向，积极培育园区特色，不断创新服务体制，完善技术开发和就业培训等配套功能，努力为项目引进、建设和企业生产经营提供优质服务。加强水、电、路、环保等基础设施建设，实行项目向园区集中，推进项目满园，提高园区项目开工投产率和土地利用率，努力把开发区（园区）建成引领经济发展的增长极。力争全年入园项目240个以上，其中荆州开发区投资过千万元的入园项目和建成项目分别达到60个以上。

着力发展产业集群。加快运用高新技术和先进适用技术改造提升传统产业，推进信息化与工业化融合，加强产业内部配套协作，延伸产业链条，不断提高产业集中度和市场竞争力。重点抓好中心城区机电汽配、纺织印染、化工医药、石油装备和造纸包装以及松滋市冶金机械、公安县汽车零部件、石首市精细化工、监利县食品加工、洪湖市石化装备等产业集群，力争重点产业集群销售收入增长20%以上。大力发展机电一体化装备制造、通讯电子、生物技术与新医药、环保节能与新材料新能源等产业，力争高新技术产业增加值增长25%。积极培育百亿元产业和企业，力争通过3-5年努力，机电、化工、纺织、轻工、农产品加工和电子信息等产业销售收入超过100亿元，1-2家企业销售收入达到100亿元、3-4家达到50亿元以上。

着力培育市场主体。继续推进“双百工程”，重点支持100家骨干企业扩产增量、提升实力，力争销售收入过亿元的企业新增5家；对100个年投资额过千万元的工业项目，实行动态管理，跟踪服务，重点抓好沙市热电联产、沙隆达吡啶、离子膜烧碱、龙江龙精密紧固件、府场化工填料和纺织印染工业园、城南浙商工业园及拍马、白云边、凯乐、金安、楚源、吉象、大枫扩规等项目建设，力争尽早建成投产。加快实施中小企业成长工程，加强服务和指导，积极引导发展“专、精、特、新”产品，确保新增规模以上企业100家以上。

着力提高企业创新能力。充分发挥企业创新主体作用，开展多种形式的产学研对接，支持骨干企业建设技术工程中心，提高自主创新和产品研发能力。落实鼓励自主创新的政策，加快科技创新与技术服务平台建设，推动科研成果向现实生产力转化。大力实施质量振兴和精品名牌工程,支持企业开展质量体系认证，力争新增中国名牌2个以上，中国驰名商标实现零的突破。加强企业家和各类技术人才的培养、引进和使用，健全收入分配、表彰奖励等激励机制，营造尊重企业家和鼓励创新的良好氛围。

（二）扎实开展新农村建设。坚持把解决“三农”问题作为重中之重，以增加农民收入为核心，突出发展现代农业，努力转变农业发展方式，力争农业总产值增长4%。

切实抓好农业结构调整。继续实施优质稻产业提升工程、畜牧业振兴工程和水产业壮大工程，积极争创畜牧大县和水产大县，推进农业区域化布局、规模化生产、产业化经营。扩大优质稻种植面积，力争优质稻面积增40万亩，粮食产量保持在70亿斤左右。大力发展养殖小区，努力扩大养殖规模，力争生猪出栏增50万头，家禽出笼增1000万只。加快实施水产“211”工程，抓好特色板块和渔业基地建设，确保水产品产量增10万吨。

切实抓好农业产业化经营。支持农业产业化龙头企业发展，鼓励龙头企业通过增资扩股、联合重组等方式，壮大规模和实力，促进农产品加工业突破性发展。推进农产品标准化生产，提高农产品质量，力争新增标志性农产品100个以上。积极培育一批规模大、实力强的农业科技服务和市场流通主体，重点办好18家省级示范性农民专业合作组织，力争新增农民专业合作社50家。加强基层农业技术推广体系建设，加快先进实用农业机械推广应用，强化疫病防控，为全市农户散养畜禽免费提供强制免疫和圈舍消毒药品。

切实抓好农村基础设施建设。实施江河崩岸整治、荆南四河堤防加固、四湖流域综合治理和水库除险工程，加快大型泵站、灌区改造步伐，力争完成水利建设土方4170万方。扎实抓好防汛抗灾工作，确保抗灾夺丰收。整合支农资金，加强通村公路、土地整理、“一建三改”和扶贫开发与老区建设。认真做好村庄规划，积极开展迁村腾地和村庄环境整治，努力改善农村卫生面貌和居住环境。扎实开展新农村建设，继续办好李埠镇、花基台等139个新农村建设试点。

切实抓好农村政策落实。加大支农惠农政策落实力度，严禁挤占、挪用和截留各类补贴资金，确保及时足额发放到位。积极做好粮食保护价收购工作。加强涉农收费和村组“一事一议”筹资筹劳监管，切实减轻农民负担。因地制宜发展村级集体经济，做好“普九”化债工作，防止新增村级债务。

（三）积极发展现代服务业。坚持以市场为导向，发挥优势，完善体制，整合资源，促进现代服务业发展提速、比重提高。

加快发展文化旅游业。制定促进旅游业发展的政策措施，按照全面开工、重点突破的要求，抓好熊家冢楚墓主车马坑的发掘与建馆开发、关公祠复建、关庙和章华寺、烈士陵园、太师渊公园整体开发等景点建设，启动荆州文保中心主体工程，积极开展荆州古城墙世界文化遗产申报工作，推动三国文化城、纪南城遗址公园以及海子湖旅游度假区建设。加大洪湖、洈水、天鹅洲等景区开发建设力度，逐步建成富有特色的旅游景区。筹建市旅游散客中心，推进涉外旅游码头建设，加强旅游宣传促销，拓展国内外旅游市场。积极培育文化市场主体，扶持壮大民营文化企业，提升传媒文博、演艺娱乐、创意策划等文化业态，繁荣文化市场。

加快发展商贸物流业。围绕建设商贸物流枢纽城市，认真实施城区商业网点规划，规范市场建设和管理，加快北京路中心商圈、两湖平原农产品交易物流中心和华中轻纺城等市场建设，努力建成现代物流园区和专业物流中心。积极适应经济转型与消费结构的新变化，运用现代经营方式改造提升商贸、餐饮等传统服务业，加快发展信息、中介等服务业，努力提升服务业发展水平。深入推进流通体制改革试点，抓好“万村千乡”市场工程，扩大农村消费。整顿和规范食品、药品、农资、建材等市场秩序，严厉打击制假售假行为。

加快发展金融服务业。积极推动国有商业银行加快发展，鼓励保险、证券等机构拓展业务，深入开展与开发银行的合作，争取更多的信贷资金支持。加快推进市商业银行增资扩股，支持农村信用社处置不良资产，增强发展后劲。积极引进境内外金融机构开设服务网点，抓好村镇银行和农业政策性保险试点。加强融资担保平台建设，完善政银企合作和考核奖励机制，扎实开展金融机构服务满园工程、产业化龙头企业和县市行活动，激励金融机构扩大信贷规模。加强诚信教育，推进信用环境综合治理，逐步建立起以个人信用为基础、企业信用为核心、政府信用为保障的社会信用体系，争创A级信用市州。

（四）进一步深化改革和扩大开放。坚持以开放促改革、促发展，全方位开展招商引资，积极承接产业转移，增强经济社会发展活力。

加大改革攻坚力度。深化农村综合配套改革，完善“以钱养事”机制；积极探索农村土地流转办法和适度规模经营，基本完成小三场改革和集体林权制度改革任务。继续深化国有企业改革，抓紧做好国有工业企业改制煞尾工作，基本完成国有“非工”企业改制任务，引导企业建立规范的现代企业制度；强化国有资产监管，促进国有资产保值增值，确保天发石油、天颐科技复牌，争取新增1-2家上市企业。深化财政体制改革，在市直全面实施规范的部门预算，实行严格的收支两条线管理，调动各方面理财的积极性。推进事业单位分类改革，逐步建立与市场经济相适应的公共事业发展体制。

加大招商引资力度。围绕主导产业、支柱企业和产业链延伸，认真策划一批促进结构优化升级的大项目。加强政府引导，坚持大员上阵，精心组织长三角、珠三角和境外专题招商活动，着力引进一批技术含量高、投资强度大、产出效益好的项目。充分发挥企业招商主体作用，改进招商方式，拓宽招商领域，实行引资与引智相结合，积极采用股权转让、资产重组、特许经营等形式，扩大职业教育、文化旅游、城市建设等投资。加强招商项目跟踪联系和协调服务，力争到位资金达到130亿元，增长30%。积极引进战略投资者和境外资本，支持外资企业增资扩股，提高利用外资水平。同时，加强大通关建设，扩大机电、化工、纺织及农产品出口规模。支持有条件的企业境外办厂、承包工程和开展农业综合开发与劳务合作。

加大对非公有制经济发展的扶持力度。鼓励和引导非公企业通过重组、兼并、联合等方式做大做强，不断提高企业素质和产业层次。按照平等准入、公平待遇原则，放宽注册资本、股东出资方式等条件，支持非公有制经济进入公用事业、基础设施、金融服务以及社会事业等领域，促进民营经济加快发展。加快中小企业服务体系建设，加大全民创业扶持力度，营造政府鼓励创业、社会支持创业、民众自主创业的良好氛围。

（五）加快基础设施建设步伐。坚持推进城镇化进程，不断完善基础设施和承载功能。

加强中心城区建设和管理。抓紧做好总规修编评审，强化规划的可操作性，严肃查处违规建设行为。进一步增强市城投公司融资和经营功能，力争完成投融资4亿元。围绕提升城市整体形象，抓好城市主要出入口和主干道改造升级，完成荆沙大道建设工程，改善道路交通条件。推进集美垃圾发电项目建设，以治理护城河为重点，改善城区水环境，促进人水和谐。坚持建设与管理并重，创新城市管理体制机制，充分发挥街道、社区参与城市管理的作用。深入开展“迎奥运圣火、创卫生城市”活动，抓好奥运火炬传递沿线街道“洗脸整容”，下大力整治乱搭乱建、乱扔乱倒、占道经营等行为，争创省级卫生城市，以文明、整洁的城市形象，迎接奥运圣火传递。规范土地出让和房地产市场管理，推动房地产业健康发展。

加强县市城区和中心镇建设。坚持“一主三化”方针，推进县市进一步扩大开放，不断壮大县域经济实力，力争“十一五”末有1-2个县市进入全省经济发展第一方阵。突出规划引导和产业兴镇，推进“百镇千村”示范工程，建设一批特色产业集镇。加强城镇道路和公用设施建设，提高产业集聚和人口吸纳等综合承载能力。

加强交通电力等重大项目建设。交通力争完成投资23亿元，建成东荆河大桥、随岳高速荆州段，加快荆监一级公路、盐卡港二期扩建步伐，争取开工建设洪监高速公路。抓好荆岳长江大桥、沪蓉高速铁路荆州段和引江济汉工程建设服务，做好江南高速公路、荆岳铁路立项争取工作。电力投资3.1亿元，开工建设220KV荆东输变电工程，完成220KV容城变电增容工程。加强重点项目协调服务，确保完成年度投资计划。

（六）切实做好增收节支工作。

积极培植税源。尊重纳税人，服务纳税人，营造税收征管的良好环境。加强烟草市场管理，扩大卷烟销售总量，支持电力、金融等税收过亿元的行业和楚源、白云边等税收过亿元的企业加快发展，壮大骨干税源。采取重点帮扶等措施，培植一批税收过千万元的利税大户，促进税收持续较快增长。开展税源普查，坚持依法治税，严格税收征管，严厉打击偷、逃、抗税行为，做到应收尽收。强化财政对税收和非税收入的监督协调，努力增加工商税收占财政收入的比重，不断提高财政收入质量。

强化支出管理。调整优化支出结构，下大力压缩非生产性和一般性支出，确保工资发放、机关正常运转、社会稳定等支出需要，提高财政保障能力。牢固树立过紧日子的思想，弘扬艰苦奋斗的优良作风，从严控制出国考察和接待费用，减少行政成本。严肃财经纪律，严格执行国库集中收付和政府采购制度，加强对转移支付和财政投资等专款的监管，提高资金使用效益。规范行政事业单位占有、使用、处置国有资产行为，加强政府债务管理，提高科学理财水平。

（七）加强资源节约和生态环境保护。坚持综合治理，标本兼治，加大统计考核和监督执法力度，落实节能减排问责制和“一票否决制”，确保完成节能减排目标任务。

狠抓节能降耗工作。积极推广节能、节材、节水新技术和新工艺，实施重点企业节能改造，推进化工、建材、造纸等高耗能行业节能降耗。采取产业链延伸、资源综合利用、废弃物集中处理、热电联供等方式，大力发展循环经济，推进清洁生产。认真执行建筑节能设计和公共建筑空调温度控制标准，促进商业和民用节能。

狠抓生态环境保护。抓好造纸、印染、化工等行业的监管，完善企业治污设施，依法关闭产能落后、污染严重的企业。认真执行环评制度，严格环保准入标准，防止新上高污染项目。积极推广生态农业模式，减少农村面源污染。加强县市城区污水处理设施建设，完善排污费核定征收管理办法，提高城市污水处理能力。加强重点区域的水资源保护和水环境治理，抓好洪湖湿地保护，通过3-5年的努力，把四湖流域建成水环境建设的示范区。

（八）加快推进以改善民生为重点的社会建设。坚持统筹发展各项社会事业，着力解决民生问题，努力促进社会和谐。

大力发展教育科技卫生等社会事业。认真落实义务教育经费保障机制，增加农村中小学公用经费，加快农村寄宿制学校和中小学改水改厕项目建设。稳步扩大优质高中教育规模，大力发展职业教育和高等教育，支持长江大学建成高水平综合性大学。努力增加科技投入，在落实政策、搭建平台、聚集人才上下功夫，加快科技中介服务和人才队伍建设，推动科技创新和科技进步。抓好农村和社区卫生服务网络建设，扩大新型农村合作医疗覆盖面，参合标准提高到100元。扎实推进血防整县推进工程，力争达到血吸虫病疫情控制标准。抓好艾滋病、结核病等疾病防控，增强公共卫生事件应急处理能力。加强基本农田保护与建设，认真执行土地管理政策，努力提高土地利用效率。扎实开展计划生育工作，稳定低生育水平。加强粮食、副食、生产资料等物价监管，努力保持居民消费价格基本稳定。认真开展第二次经济普查。积极发展残疾人和老龄事业，中心城区实行65岁以上老年人免费乘坐公交车。做好民族宗教、台湾事务、外事侨务、档案、地方志、地震等工作。

切实抓好就业和社会保障工作。扎实推进统筹城乡就业试点工作，鼓励自主创业、自谋职业和灵活就业，帮助零就业家庭、就业困难人员就业，做好大中专毕业生和转业退伍军人就业工作，力争全年新增城镇就业7.5万人。规范企事业单位用工行为，切实维护劳动者合法权益。继续做好国有困难企业退休人员和城镇低保人员参加医疗保险工作，开展城郊失地农民基本养老保险试点，抓好非公有制企业、灵活就业人员参加基本养老保险和续接工作，力争扩面新增9.6万人次，征收社会保险费16.8亿元。规范城乡低保管理，完善大病医疗和特困户救助制度，切实做好优抚安置、五保户集中供养等工作。建立健全经济适用房和廉租房制度，努力解决低收入家庭住房困难。

加强社会主义精神文明建设。加强中国特色社会主义核心价值观教育，推进文化、科技、法律、卫生“四下乡”、“四进社区”工作，加强青少年思想道德教育，深入开展文明城市、文明村镇、文明行业、文明单位创建活动，培育文明和谐新风尚。积极发展新闻出版、文化体育、广播电视事业，重视文物和非物质文化遗产保护，加强基层文体活动场所和有线电视“村村通”工程建设，广泛开展“全民健身与奥运同行”活动，丰富群众精神文化生活。

切实加强基层民主建设，保障人民的知情权、参与权、表达权、监督权。加强农村基层组织建设，完成村委会换届选举工作。加强村（居）办公场所建设，建立健全以村（居）民代表大会为基本形式的民主管理制度，推进村（居）务公开，增强自治和服务功能。

高度重视社会稳定工作。加强社会治安综合治理，严厉打击犯罪行为，推进平安荆州建设。深入开展“五五”普法，抓好法律援助、人民调解、社区矫正等工作。强化信访工作责任制，妥善解决群众的合理诉求，把矛盾化解在基层。健全应急管理机制，提高处置公共突发事件能力。强化安全生产责任，预防重特大安全事故发生。同时加强国防教育、国防动员、预备役部队和民兵建设、双拥和人防工作。

努力办好十件实事。（1）培训农村劳动力4万人，新增转移农村劳动力8万人；帮助3000人实现创业并带动1万人就业再就业。（2）继续实施血防整县推进和四湖流域综合治理工程，解决农村45.9万人的饮水安全问题，新建沼气池6万口，免费救治晚血病人2600人；建设四湖流域12个乡镇污水处理项目。（3）实现新型农村合作医疗制度全覆盖，参合率达到80%以上，住院医疗费用补偿率不低于30%；建设200个标准化村卫生室。（4）免除城乡义务教育阶段学生的学杂费，免费为农村义务教育阶段的学生提供教科书，提高农村义务教育阶段家庭经济困难寄宿生的生活费补助标准。（5）完善农村最低生活保障制度，将农村特困救助对象扩大到14万人。（6）新建通村公路1000公里，新增通油路、水泥路的行政村400个，完成渡口维修改造96处，新建农村公路候车亭180个；新建或改造标准化农家店700家。（7）黑化升级城区南环路、郢都路、荆东环路，完成城南、草市污水处理厂建设，疏挖和整治荆沙河、荆襄河。（8）城区建设经济适用房8万平方米，抓紧做好已开工的危旧房拆迁建设工程，启动新沙路、中山路西片危改项目，对人均住房面积不足10平方米的最低收入家庭实行住房援助。（9）建立市级法律援助远程网上申请、受理、咨询系统，建设标准化基层法律援助工作站80个。（10）免费为100名贫困下肢残疾人安装假肢、为100名贫困白内障患者实施复明手术；为100户农村贫困残疾人实施危房改造，每户补助资金5000元。

三、加强政府自身建设

新形势、新任务对政府自身建设提出了新的更高要求。我们将切实转变政府职能，坚持依法行政，加强管理创新，努力建设服务型政府。

坚持依法行政。认真贯彻落实人大及其常委会的决议、决定，自觉接受人大法律监督、政协民主监督，认真听取各民主党派、工商联、无党派人士和各人民团体的意见，虚心接受新闻媒体和社会公众监督，及时报告、通报、发布政府工作情况。认真办理人大代表建议、批评、意见和政协委员提案，不断改进政府工作。加强行政执法队伍建设，规范执法行为，切实解决多头执法、重复执法和乱收费、乱罚款问题。健全行政执法责任追究制度，加强行政复议工作，提高依法行政水平。

推进管理创新。树立服务第一的观念，加快转变政府职能，推进政事、政企分开，中介机构与部门脱钩，强化社会管理和公共服务，让职责、权力服从服务于发展的需要，让规矩、程序服从服务于效率的需要。进一步清理行政审批事项，减少收费项目，降低收费标准，推进行政审批向部门内设机构集中，部门审批向行政服务中心集中，实施联审联办、全程代办和网上审批，提高审批服务效率。创新公众参与、专家论证和政府决策相结合的决策机制，提高科学决策水平。深入推进政务公开，加强电子政务和政府网站建设，完善新闻发布和“市长信箱”工作机制，增强政府工作的透明度。

转变工作作风。牢固树立科学发展观和正确的政绩观，大力弘扬求真务实、埋头苦干的作风，坚持重实际，说实话，办实事，求实效，把抓发展落实到产业、项目和企业上来，落实到为市场主体创造良好环境上来。加强热点、难点问题的调查研究，增强工作的预见性、主动性和创造性。切实精减会议、文件和各类检查评比活动，集中精力抓改革、谋发展、促稳定。建立健全抓落实的工作机制，完善目标管理、政务督查和绩效考核，确保政令畅通和各项工作落到实处。

加强廉政建设。加强政府系统工作人员理想信念和廉洁从政教育，强化宗旨观念，努力提高公务员队伍整体素质。围绕规范行政权力运行，推进体制改革和制度建设，拓宽源头治腐领域，努力形成靠制度管人、制权、理事的有效机制。认真落实党风廉政建设责任制，严格执行工程建设招投标、经营性土地使用权招拍挂等制度，加强行政监察和审计监督，坚决纠正损害群众利益的不正之风，严肃查处违纪违规行为，树立人民政府的良好形象。

各位代表，做好今年的各项工作，任务艰巨。让我们紧密团结在以胡锦涛同志为总书记的党中央周围，在省委、省政府和市委的领导下，团结拼搏，开拓创新，为开创全面建设小康社会新局面而努力奋斗！
